# Cerebral palsy in art and literature throughout history

**DOI:** 10.1055/s-0046-1817030

**Published:** 2026-02-27

**Authors:** Patricia do Rocio Litça, Ana C. de Souza Crippa, Adrielle Holler Pykocz, Luis F. Fabrini Paleare, Marcio Vieira Sanches Silva, Filipe M. Barcelos, Helio A. G. Teive, Gustavo Leite Franklin

**Affiliations:** 1 Universidade Federal do Paraná, Hospital das Clínicas, Unidade de Neuropediatria, Curitiba PR, Brazil.; 2 Pontifícia Universidade Católica do Paraná, Escola de Medicina, Departamento de Medicina Interna, Curitiba PR, Brazil.; 3 Hospital Estadual Infantil Nossa Senhora da Glória, Departamento de Ortopedia e Traumatologia, Vitória ES, Brazil.; 4 Hospital Israelita Albert Einstein, São Paulo SP, Brazil.; 5 Universidade Federal do Paraná, Hospital de Clínicas, Departamento de Medicina Interna, Serviço de Neurologia, Curitiba PR, Brazil.; 6 Universidade Federal do Paraná, Hospital de Clínicas, Departamento de Medicina Interna, Curitiba PR, Brazil.

**Keywords:** Cerebral Palsy, History of Medicine, Art, Literature

## Abstract

Cerebral palsy (CP), a term coined by William John Little in 1843, represents a group of non-progressive motor disorders resulting from early brain injury. Beyond its medical characterization, there were early artistic depictions, such as Egyptian reliefs and medieval religious scenes, portraying individuals with asymmetric or contracted limbs mainly through symbolic or moral lenses. During the Renaissance and Baroque periods, artists including Dürer and Velázquez subtly represented physical diversity, though without explicit medical context. Literary portrayals evolved from mythological or moral allegory (e.g., Hephaestus, hagiographies) to empathetic narratives of individuality and inclusion, as seen in Tiny Tim, present in *A Christmas Carol,* written by Charles Dickens, and Draper's *Out of My Mind* . In modern times, artists and writers living with CP transformed disability into a means of self-expression and social critique.

## INTRODUCTION

The term *Cerebral Palsy* (CP) was coined by William John Little, an English orthopedist, in 1843, to describe a non-progressive condition characterized by muscular rigidity. 1 Although no explicit artistic records were labeled as CP before the 19th century, depictions of motor impairments appear in paintings, sculptures, and texts throughout history. From ancient Egypt to modernity, these portrayals reveal both clinical observations and symbolic-cultural meanings. 2 3 4 5 In contemporary times, artists and writers with CP themselves provide authentic perspectives, bridging disability, creativity, and inclusion. 6 7

## CEREBRAL PALSY IN ART

**Figure 1:**
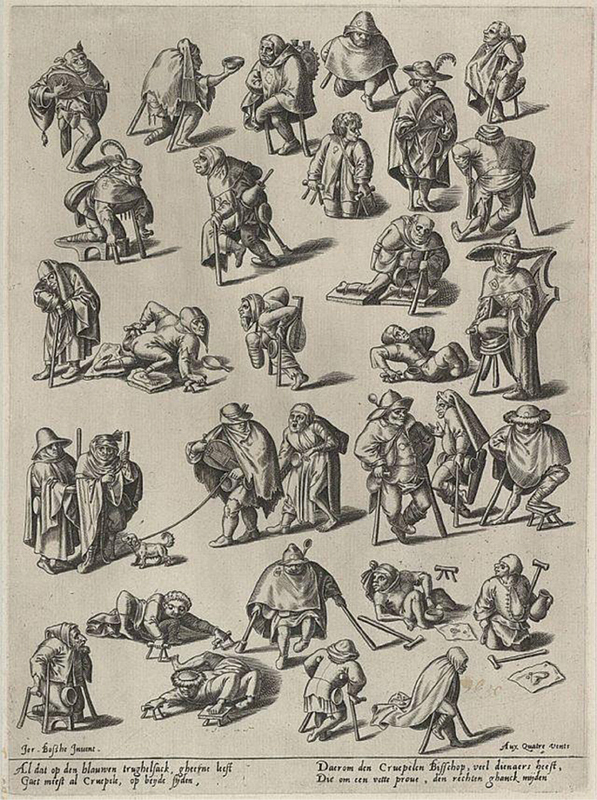
This pen-and-ink drawing depicts a satirical procession of impoverished beggars, fools, and disabled figures—some limping on crutches with twisted limbs or playing crude instruments—evoking themes of social marginalization and divine judgment in late medieval society, where physical deformities symbolized moral or spiritual failings, rendered with Bosch's characteristic intricate detail and grotesque humor. **Source** : Public domain photograph of nineteenth-century woodblock print.

In ancient Egypt, some depictions in reliefs and paintings show individuals with asymmetrical postures or stiffened limbs, which may be interpreted as signs of neurological conditions, including CP. 2 4 In Christian medieval art, individuals with disabilities are often represented in religious contexts, such as healing miracle scenes. Sacred manuscripts and church paintings portrayed people with contracted limbs or abnormal postures. However, these images usually served moral or religious purposes rather than medical ones. A notable example is the painting *The Cripples* , created around 1500 by the Dutch painter Hieronymus Bosch and currently housed in the Museum Boijmans Van Beuningen, in Rotterdam. In this work, Bosch depicted figures with restricted movement and physical deformities, possibly inspired by real-life observations of disability ( Figure 1 ). 4 5 8

During the Renaissance, the artist Albrecht Dürer produced anatomical drawings of physically impaired figures, sometimes suggesting spasticity reminiscent of CP. 4 7 In the Baroque painting period, Diego Velázquez portrayed individuals with visible disabilities in the Spanish court. While these figures likely had other conditions (e.g., achondroplasia), Velázquez's sensitivity to physical differences suggests that conditions like CP may have also been observed. 4 5

With medical advances in the 19th century, CP began to be recognized as a distinct condition, thus leading to more conscious artistic representations. 9 10 11 Little's work distinguished CP from other childhood conditions and established its neurological nature, and, later, Osler and Freud further elaborated these concepts within neurology. 12 13 14

In the 21st century, many individuals with CP began to redefine representation by speaking and creating for themselves. Lucy Jones, a British painter living with CP uses her vivid, humorous, and introspective art to explore disability, aging, and self-image with striking honesty. Also, Dan Keplinger, an American artist best known as the subject of the Oscar-winning documentary *King Gimp* (2000), transforms his lived experience with CP into powerful expressions of perseverance and identity. Lucille Wallenrod (1918–1998) was an American modernist painter from Brooklyn, who overcame physical limitations by inventing a special arm support that allowed her to create dynamic seascapes, portraits, and still lifes distinguished by bold color and movement. María Dolors Vázquez Aznar (1955–2014), a Spanish realist painter and lawyer from Valencia, taught herself to paint with her left foot and pursued a professional artistic career while also championing disability rights through her leadership in the Christian Fraternity of People with Disabilities (FRATER). Collectively, their lives and work challenge societal perceptions, transforming disability into a source of artistic strength and human insight.

## CEREBRAL PALSY IN LITERATURE

In Homer's *Iliad* , Hephaestus, god of fire, and metallurgy, is described as “lame,” with “shrunken” or “twisted” feet, weak legs, and a limping gait, potentially a literary representation of CP or other similar condition. Despite this, his arms and torso are strong and robust, allowing him to create masterpieces, such as the armor of Achilles. Such portrayals carried symbolic weight, associating disability with resilience. 4

In medieval European literature, similar to art, characters with physical disabilities frequently appear in religious or allegorical contexts. Hagiographies (lives of saints), for example, often describe “miraculous cures” involving people referred to as “paralytics” or with contracted limbs. 4 5 15 One such example is the *Life of Saint Cuthbert* (7th century), which mentions individuals with motor difficulties—potentially compatible with CP—but interpreted through a spiritual lens as “divine punishment” or illness to be healed. In Geoffrey Chaucer's *The Knight's Tale* ( *The Canterbury Tales* , late 14th century), there are mentions of characters with physical frailty, but no details sufficient to suggest CP specifically. 4 5 15

During the Renaissance, literature began portraying the human condition more realistically, though direct references to CP remain rare or ambiguous. In *Gargantua and Pantagruel* (1532–1564) by Rabelais, characters with unusual physical features are described, but often exaggerated for satire rather than realism or clinical accuracy. 8 16

In the 18th century, during the Enlightenment, interest in medical descriptions grew. Medical texts by authors like William Cullen began mentioning cases of “infantile paralysis,” but fictional literature continued to portray disabilities in stereotyped ways—for instance, the “crippled” characters in folk tales or moral fables. 8

**Figure 2:**
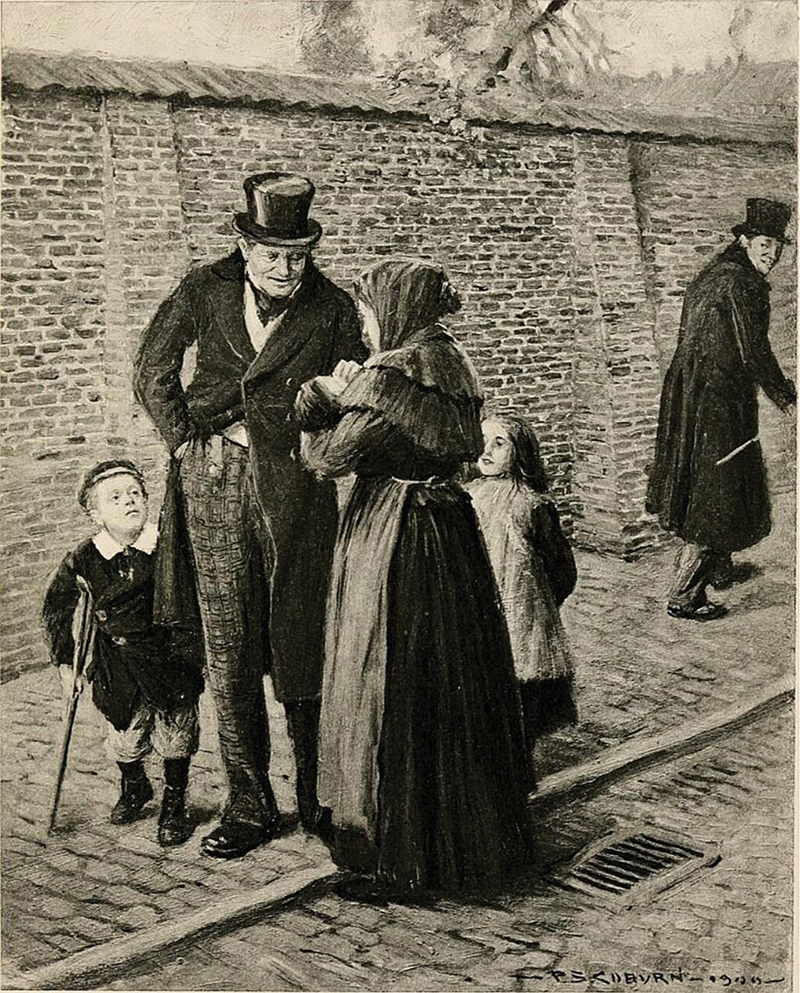
Tiny Tim, fragile in appearance and holding his small crutch. *A Christmas Carol* (1900), illustration by Frederick Simpson Coburn. **Source** : Internet Archive Book Images. Public domain.

In the 19th century, in Dickens's *A Christmas Carol* (1843), Tiny Tim's mobility limitations have been associated with CP ( Figure 2 ). 1 4 Also, Victor Hugo's Quasimodo in *The Hunchback of Notre-Dame* (1831) exemplifies social stigma toward disability, though without neurological accuracy. 4

The 20th century marked the rise of first-person accounts. Irish writer Christy Brown, who had CP, used his left foot to write and paint. His autobiography *My Left Foot* (1954) was later adapted into an Oscar-winning film, providing one of the first direct testimonies of living with CP. 4 7 Frances Hodgson Burnett's *The Secret Garden* (1911) portrayed Colin, a child with motor weakness suggestive of neurological disease, though resolved allegorically. 5

In contemporary literature, CP is represented through narratives promoting inclusion and authenticity. *Out of My Mind* (2010), by Sharon M. Draper, tells the story of Melody, a girl with CP who cannot speak or move easily but has a brilliant mind. The book is widely praised for its realistic and empathetic portrayal. 7 In the same way, Anne Finger's *Call Me Ahab* (2009) challenges stereotypes through stories centering on disability identity. 7

Although clarity in historical representations of CP may be challenging, due to the wide spectrum of its clinical manifestations, many descriptions across centuries in literature and art highlight the enduring presence and significance of this condition in human society. In recent times, growing clinical understanding and social awareness have helped refine its definition, reduce misconceptions, and gradually dismantle long-standing stigma.
